# Poisoned Air

**Published:** 1891-06

**Authors:** 


### POISONED AIR.

When we breathe over and over again the same air we gradually vitiate it by the constant exhalation of carbonic acid, which gradually brings the air up to the point where the difference between it and the blood—as regards the proportions of carbonic acid—disappears. The blood ceases to be arterialized, and the vital functions are arrested. In vain does the air still contain a quantity of life-giving oxygen ; the blood cannot take it up, because it cannot get rid of its carbonic acid ; the conditions of exchange are absent. To place an animal in air overcharged with carbonic acid is equivalent to a gradual prevention of his breathing at all. Suffocation results from vitiation of the air, in precisely the same manner as from interception of the air.

Professor Brown-Sequard has recently been making experiments to determine whether the human breath was capable of producing any poisonous effects. From the condensed watery vapor of the expired air he obtained a poisonous liquid which, when injected under the skin of rabbits, produced almost immediate death. He ascertained that this poison was an alkaloid, and not a microbe. The rabbits thus injected died without convulsions, the heart and large blood vessels being engorged with blood. M. Brown-Sequard considers it fully proved that the expired air, both of man and animals, contains a volatile poisonous principle which is much more deleterious than the carbonic acid it contains.

In the reign of King George II., the Rajah of Bengal took some English prisoners in Calcutta and put 146 of them into a place which has been known since the Indian Mutiny as the "Black Hole." This place was only 18 feet square by 16 feet high, and ventilation was provided for only by two small grated windows out of reach of all. One hundred and twenty-three of the prisoners died in the night, and most of the survivors were afterwards carried off by putrid fever, due to the fetid atmosphere caused by so many human breaths.

